# Look at the patient—in sugar and infection

**DOI:** 10.1186/s13054-015-1176-2

**Published:** 2015-12-30

**Authors:** Sascha Tafelski, Claudia Spies, Irit Nachtigall

**Affiliations:** Department of Anaesthesiology and Intensive Care, Charité-Universitaetsmedizin Berlin, Campus Charité Mitte and Campus Virchow-Klinikum, Augustenburger Platz 1, 13353 Berlin, Germany

With great interest, we followed the recent publication [1] regarding glucose management. The observational study evaluates patients in a medical-surgical intensive care unit (ICU) and reports on associations between time in targeted blood glucose range (TIR) and ICU mortality. The authors defined a TIR of 70–140 mg/dl (local hospital policy). The observed association with mortality was limited to non-diabetic patients and this is highly relevant. The presence of diabetes seems to reduce a protective effect of intensive blood glucose management in non-diabetics. A protective effect related to an intrinsic non-glucose-regulating mechanism of insulin [2, 3] was discussed but could also be altered by insulin resistance. Here, HBA1c measured on ICU admission or other surrogates (e.g., C-peptide related to blood glucose) could serve as potent biomarkers to identify patients who may benefit from glucose management. Every observation is exploratory and it is impossible to draw conclusions on causality. Other interacting factors like quality of infection management are also strongly related to ICU survival [4], and patients with diabetes have an increased risk to develop severe infections [2]. Interestingly, the findings of Krinsley and Preiser [1] are in line with results from another study in surgical ICU patients [5]. When slightly different cutoffs for low TIR were used, this measurement was associated with ICU mortality (odds ratio = 3.69, *P* = 0.013) for patients with lower achieved quality in blood glucose management [5].

In contrast to most published guidelines, future recommendations may need to include individualized algorithms (e.g., for patients with and patients without diabetes mellitus) [2]. In this context, we agree with the authors that a single target range of blood glucose management in the ICU setting seems to be arbitrary and further studies are required to study individualized therapy algorithms in both the surgical and the non-surgical ICU setting.

## Abbreviations

ICU: intensive care unit
TIR: time in targeted blood glucose range
